# Micromotor-enabled active drug delivery for in vivo treatment of stomach infection

**DOI:** 10.1038/s41467-017-00309-w

**Published:** 2017-08-16

**Authors:** Berta Esteban-Fernández de Ávila, Pavimol Angsantikul, Jinxing Li, Miguel Angel Lopez-Ramirez, Doris E. Ramírez-Herrera, Soracha Thamphiwatana, Chuanrui Chen, Jorge Delezuk, Richard Samakapiruk, Valentin Ramez, Marygorret Obonyo, Liangfang Zhang, Joseph Wang

**Affiliations:** 1 Department of NanoEngineering, University of California San Diego, La Jolla, CA 92093 USA; 2 Department of Medicine, University of California San Diego, La Jolla, CA 92093 USA

## Abstract

Advances in bioinspired design principles and nanomaterials have led to tremendous progress in autonomously moving synthetic nano/micromotors with diverse functionalities in different environments. However, a significant gap remains in moving nano/micromotors from test tubes to living organisms for treating diseases with high efficacy. Here we present the first, to our knowledge, in vivo therapeutic micromotors application for active drug delivery to treat gastric bacterial infection in a mouse model using clarithromycin as a model antibiotic and *Helicobacter pylori* infection as a model disease. The propulsion of drug-loaded magnesium micromotors in gastric media enables effective antibiotic delivery, leading to significant bacteria burden reduction in the mouse stomach compared with passive drug carriers, with no apparent toxicity. Moreover, while the drug-loaded micromotors reach similar therapeutic efficacy as the positive control of free drug plus proton pump inhibitor, the micromotors can function without proton pump inhibitors because of their built-in proton depletion function associated with their locomotion.

## Abstract (web summary)

Nano- and micromotors have been demonstrated in vitro for a range of applications. Here the authors demonstrate the in-vivo therapeutic use of micromotors to treat *H. pylori* infection.

## Introduction

Recent advances in the nano and micromotor field^1–4^ in terms of improvement of biocompatibility and biological function have led to their growing use in biomedicine^5–7^, including therapeutic payload delivery^8–13^, micro-surgery^14, 15^, isolation of biological targets^16^, operation within living cells^17, 18^, and removal of toxicant molecules and organisms^19–21^. Although significant progress has been accomplished to demonstrate the in vitro capabilities of nano/micromotors to transport therapeutic cargos to target destinations, tremendous effort is still required to translate the proof-of-concept research to in vivo biomedical applications.

In recent years, the utility and performance of these motor-based active transport systems have been tested in live animals. For example, our group has demonstrated the attractive in vivo performance of zinc-based and magnesium (Mg)-based micromotors under in vivo conditions^22–24^. These studies have shown that artificial micromotors can self-propel in the stomach, and intestinal fluids for enhanced retention in the gastric mucous layer^22^ and targeted delivery in the gastrointestinal (GI) tract^23^. Walker et al.^25^ presented the ability of magnetic micropropellers to move through gastric mucin gels, by mimicking the mucus penetration strategy of *Helicobacter pylori* (*H. pylori*). In addition, Nelson’s group has demonstrated that magnetically actuated microswimmers can swarm in vivo^11^, whereas Martel’s group has shown that microorganisms can be transformed into natural robots under magnetic guidance towards therapeutic cargo delivery into deep tumor regions^12^. These prior in vivo studies of synthetic motors have significantly advanced motor research and cleared a path towards direct evaluation of disease-oriented therapeutic efficacy associated with motor-enabled active drug delivery. However, this still remains an alluring but unmet goal for biomedical researchers.

This work demonstrates, to the best of our knowledge, the first attempt to apply Mg-based micromotors, loaded with antibiotic drug clarithromycin (CLR), for in vivo treatment of *H. pylori* infection in a mouse model. Given the built-in proton depletion function, this motor-based therapy is able to undergo the harsh gastric environment to achieve antibacterial efficacy without involving the commonly used proton pump inhibitors (PPIs). The *H. pylori* bacteria, found in about half of the world’s population, can cause stomach infection and subsequently lead to diverse gastric and extragastric diseases^26, 27^. In most cases, the administration of antibiotics for the treatment of *H. pylori* infection is combined with the use of PPIs to reduce the production of gastric acid^28^, because the gastric acid could make antibiotics less effective. The effectiveness of PPIs is attributed to the irreversible binding to proton pumps and thus to suppress acid secretion^29, 30^, which in long term use can lead to adverse effects such as headache and diarrhea and in more serious scenarios cause anxiety or depression^31–34^. Therefore, it would be highly beneficial to develop an alternative therapeutic regimen with equivalent or advantageous therapeutic efficacy as the current antibiotic treatments while excluding the use of PPIs.

The reported Mg-based micromotors rely on the combination of a CLR-loaded poly(lactic-co-glycolic acid) (PLGA) layer and a chitosan polymer layer covering on a propellant Mg core to offer high drug-loading capacity, along with biodegradability. The positively charged chitosan outer coating enables adhesion of the motor onto the stomach wall^35^, facilitating efficient localized autonomous release of CLR from the PLGA polymer coating. In contrast to acid suppression by PPIs, Mg-based micromotors can temporally and physically alter the local acidic environment by quickly depleting protons while propelling within the stomach^24^. By using acid as fuel, these synthetic motors rapidly deplete protons while propelling within the stomach, which can effectively elevate the gastric pH to neutral in < 20 min after the motors are applied^24^. Testing in a mouse model has demonstrated that these motors can safely and rapidly neutralize gastric acid without causing noticeable acute toxicity or affecting the stomach function, and that the normal stomach pH can be restored within 24 h post motor administration. Such elimination of the PPI administration is coupled with significant reduction of bacteria burden, as demonstrated in vivo in a mouse model. Using a mouse model of *H. pylori* infection, the propulsion of the drug-loaded Mg-based micromotors in gastric fluid along with their outer chitosan layer are shown to greatly enhance the binding and retention of the drug-loaded motors on the stomach wall. As these micromotors are propelled in the gastric fluid, their Mg cores are dissolved, leading to self-destruction of these motors without harmful residues, as is demonstrated by the toxicity studies.

Overall, we take advantage of the efficient propulsion of Mg-based micromotors in the acidic stomach environment, their built-in proton depletion ability, their active and prolonged retention within the stomach wall, and their high drug-loading capacity, to demonstrate to the best of our knowledge the first actual in vivo therapeutic application of chemically powered micromotors. In vivo studies examine the therapeutic efficacy, distribution, and retention of the micromotors in the mouse stomach compared with passive drug-loaded microparticles and other control groups, along with the corresponding in vivo toxicity profile. These results illustrate the attractive therapeutic capabilities of acid-driven micromotors, which open the door for in vivo therapeutic applications of body-fluid propelled micromotors towards the treatment of a variety of diseases and disorders.

## Results

### Drug-loaded Mg-micromotors preparation and characterization

**Fig. 1:**
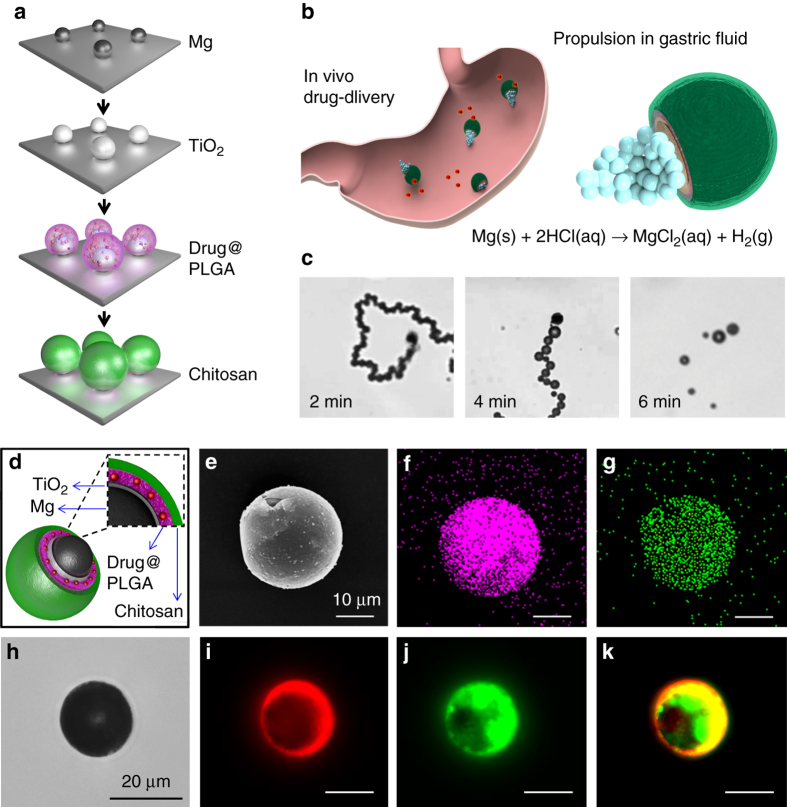
Synthesis and characterization of drug-loaded Mg-based micromotors. **a** Schematic preparation of the micromotors: Mg microparticles dispersion over a glass slide, TiO_2_ atomic layer deposition (*ALD*) over the Mg microparticles, drug-loaded PLGA deposition over the Mg-TiO_2_ microparticles, and Chitosan polymer deposition over the Mg-TiO_2_-PLGA microparticles. **b** Schematic of in vivo propulsion and drug delivery of the Mg-based micromotors in a mouse stomach. **c** Time-lapse images (2 min intervals, taken from Supplementary Movie 3) of the propulsion of the drug-loaded Mg-based micromotors in simulated gastric fluid (pH ~1.3). **d** Schematic dissection of a drug-loaded micromotor consisting of a Mg core, a TiO_2_ shell coating, a drug-loaded PLGA layer, and a chitosan layer. **e** Scanning electron microscopy (*SEM*) image of a drug-loaded Mg-based micromotor. **f**, **g** Energy-dispersive X-ray spectroscopy (*EDX*) images illustrating the distribution of **f** magnesium and **g** titanium in the micromotor. **h**–**k** Microscopy images of dye-loaded Mg-based micromotor: **h** optical image and fluorescence images showing the dye-loaded Mg-based micromotors in the **i** DiD channel (PLGA layer), **j** FITC channel (chitosan layer), along with an overlay of the two channels **k**

Figure 1a and Supplementary Movie 1 schematically illustrate the preparation steps of the drug-loaded Mg-based micromotors. The cores of the micromotors are made of Mg microparticles with an average size of ~20 µm. In the study, a layer of Mg microparticles was dispersed onto a glass slide, followed by an asymmetrical coating of the microspheres with a thin TiO_2_ layer using atomic layer deposition (ALD). The ALD process leads to a TiO_2_ uniform coating over the Mg-microspheres, while leaving a small opening (essential for contact with the acid fuel) at the sphere-glass contact point^36^, which forms a Janus microstructure. Such TiO_2_ layer acts as a shell scaffold that maintains the micromotor spherical shape and the opening size during the propulsion, leading to consistent and prolonged operation. The Mg-TiO_2_ Janus microparticles were then coated with a PLGA film containing the CLR antibiotic payload. After the drug-loading step, the microparticles were coated with an outer thin chitosan layer (thickness ~100 nm) that ensures efficient electrostatic adhesion of the micromotors to the mucosal layer on the stomach wall while protecting the CLR-loaded PLGA layer. Finally, the resulting CLR-loaded Mg-based micromotors were separated and collected by soft mechanical scratching of the glass slide, leaving a small opening for spontaneous Mg-acid reaction when the motors are placed in an acidic solution. This reaction generates hydrogen microbubbles and leads to efficient propulsion in the stomach fluid^24^. The small opening enables also a slow reaction process and gradual dissolution of the Mg core, leading to a prolonged micromotor lifetime of ~6 min. The in vivo self-propulsion in the gastric fluid of a stomach and the corresponding drug delivery process from the PLGA layer of the Mg-based micromotors are illustrated schematically in Fig. 1b and Supplementary Movie 2.

The ability of drug-loaded Mg-based micromotors to efficiently propel in gastric acid was first tested in vitro by using a simulated gastric fluid (pH ~1.3). The microscopic images in Fig. 1c (taken from Supplementary Movie 3 at 2 min intervals) illustrate the fast and prolonged autonomous propulsion of a CLR-loaded Mg-based micromotor in the gastric fluid simulant. The efficient hydrogen bubble generation propels the micromotors rapidly, with an average speed of ~120 μm s^−1^ (corresponding to a relative speed of 6 body length s^−1^), and indicates that the Mg-based micromotors can react and move fast in the gastric fluid. Such efficient micromotor propulsion is essential for the motors to reach stomach wall and thus achieving significant therapeutic efficacy. Importantly, the acid-Mg reaction responsible for the autonomous propulsion also spontaneously depletes protons in gastric fluid and thus neutralizes the stomach pH without using PPIs^24^.

Figure 1d schematically illustrates the structure of a drug-loaded Mg-based micromotor, showing the Mg core, covered mostly with the TiO_2_ shell layer, drug-loaded PLGA layer, and an outer chitosan layer. The drug-loaded Mg-based micromotors were carefully characterized. The scanning electron microscopy (SEM) image of a drug-loaded micromotor (shown in Fig. 1e) confirms the presence of a small opening (~2 µm) on the spherical micromotor, produced during the coating process, that exposes the Mg core of the micromotor to the gastric fluid and facilitates the hydrogen bubble thrust. Energy-dispersive X-ray (EDX) spectroscopy mapping analysis was carried out to confirm the motor composition. The resulting EDX images, shown in Fig. 1f and g, illustrate the presence and distribution of magnesium and titanium, respectively.

A fluorescence study was carried out to confirm efficient drug-loading within the PLGA layer, and the coating of the micromotor with the protective and adhesive chitosan layer. This was accomplished by preparing Mg-based micromotors with the PLGA and chitosan coatings containing the fluorescent dyes 1,1′-dioctadecyl-3,3,3′,3′-tetramethylindodicarbocyanine, 4-chlorobenzenesulfonate salt (DiD, *λ*_em_ = 665 nm), and fluorescein isothiocyanate-dextran (FITC, *λ*_em_ = 520 nm), respectively. An optical image of a dye-loaded micromotor is displayed in Fig. 1h. The corresponding fluorescence images show the dye-loaded Mg-based micromotor in the DiD and FITC channels (Fig. 1i and j, respectively); an overlay of the two channels is displayed in Fig. 1k. The high-fluorescent intensity of the loaded dyes confirms the successful coating of the micromotor with both PLGA and chitosan layers, along with the high cargo-loading capacity of the micromotor.

Prior to in vivo therapeutic application of the Mg-based micromotors, several in vitro studies were performed. Initially, the ability of drug-loaded micromotors to efficiently propel in gastric acid was tested in vitro. Supplementary Fig. 1a–d displays time-lapse images (corresponding to Supplementary Movie 4) showing the motion of the drug-loaded Mg-based micromotors in simulated gastric fluid adjusted to different pH values (0.75, 1.25, 1.5, and 1.75, respectively). Time-lapse images in Supplementary Fig. 1e–h show the lifetime of a drug-loaded micromotor in gastric fluid simulant (pH ~1.3) to be ~6 min. Supplementary Fig. 1i displays the pH-dependent speed of the micromotor in the gastric fluid simulant. The micromotor speed drastically decreases upon changing the pH of the gastric fluid solution from pH 1.5–1.75. Assuming that the stomach pH is 1.3, the drug-loaded Mg-based micromotors can efficiently move at this condition with an average speed of ~120 μm s^−1^ (~6 body length s^−1^).

### Drug-loading optimization and in vitro bactericidal activity

**Fig. 2:**
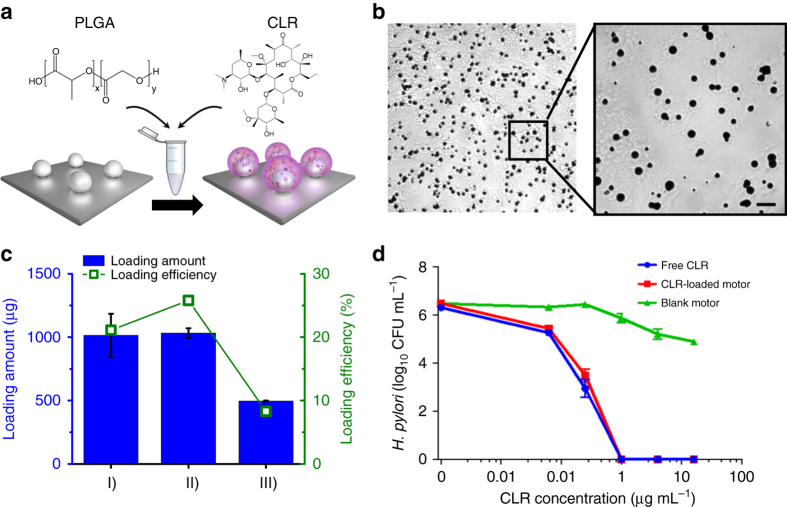
Antibiotic drug loading of the Mg-based micromotors and in vitro bactericidal activity. **a** Schematic displaying the loading clarithromycin (*CLR*) onto the Mg-based micromotors. PLGA polymer dissolved in ethyl acetate is mixed with CLR, and the solution is deposited over the Mg-TiO_2_ microparticles resulting in the formation of a thin PLGA-CLR coating. **b** Microscope images showing the PLGA-CLR film over the Mg-based micromotors. *Scale bars* 100 µm and 40 µm, respectively. **c** Quantification of CLR-loading amount and yield of the micromotors prepared with different CLR solutions: (I) 100 µL of 40 mg mL^−1^ CLR solution, (II) 120 µL of 40 mg mL^−1^ CLR solution, and (III) 200 µL of 30 mg mL^−1^ CLR solution. All the CLR-loaded Mg-based micromotors were coated with a thin chitosan layer; all samples were dissolved in acid for 24 h before the drug-loading measurement. **d** In vitro bactericidal activity of free CLR, CLR-loaded Mg-based micromotors, and blank Mg-based micromotors (without CLR drug) against *H. pylori* bacteria. *Error bars* estimated as a triple of s.d. (*n* = 3)

The CLR-loading onto the Mg-based micromotors was optimized to achieve a clinically relevant therapeutic concentration of the drug (15–30 mg kg^−1^ day^−1^)^37^. Figure 2a shows a schematic displaying the loading of CLR onto the micromotors. Briefly, the Mg-TiO_2_ microparticles dispersed onto a glass slide (~2 mg of Mg microparticles per glass slide) were coated with a PLGA solution prepared in ethyl acetate, which was mixed with CLR (see detailed experimental protocol in “Methods” section). Rapid evaporation under nitrogen current leads to the formation of a homogeneous PLGA-CLR coating over the Mg-TiO_2_ microparticles (microscope images of the coated micromotors are displayed in Fig. 2b). The microparticles were further coated with chitosan before quantifying the CLR-loading efficiency of the micromotors. To optimize the drug-loading, Mg-based micromotors were coated with PLGA solutions containing different amounts of CLR (between 4 and 6 mg). By studying different combinations of the PLGA-CLR solution volume and CLR concentration, the highest CLR-loading efficiency (26%), corresponding to 1032 ± 37 µg per 2 mg micromotor, was obtained when coating the microparticles with 120 µL of the PLGA solution containing 4.8 mg of CLR (Fig. 2c, II). This formulation offered optimal CLR-loading and was selected for subsequent in vitro and in vivo anti-*H. pylori* studies.

Once confirmed that the micromotors were capable to load antibiotic cargo with high-loading efficiency, an in vitro bactericidal activity of CLR-loaded Mg-based micromotors against *H. pylori* was performed. To mimic the gastric environment, samples were treated in 0.1 N HCl for 1 h prior to incubation with bacteria. This also ensured the dissolution of micromotors and consecutive drug release. Figure 2d shows the enumerated amount of bacteria after being treated by CLR-loaded Mg-based micromotors or free CLR solution with varying concentrations of CLR. According to the results, drug-loaded micromotors exhibited a comparable bactericidal activity to free drug solution over the whole range of concentrations used in the study. Specifically, we determined the minimal bactericidal concentration (MBC) values of the samples, defined as the minimal concentration of an antimicrobial agent that kills 3 logs (99.9%) of the bacteria. The MBC value for CLR-loaded Mg-based micromotors was found to be 0.25 μg mL^−1^, which was unaltered from the MBC value of free CLR. Moreover, bare Mg-based micromotors, with corresponding amount of motors and treated under the same conditions as the free CLR and CLR-loaded Mg-micromotors, were used as negative controls. From Fig. 2d, the bare motors had negligible effect on the viability of *H. pylori* over the studied range, which supports that the bactericidal effect of CLR-loaded Mg-based micromotors is solely due to the loaded antibiotics, and not due the other compositions of the micromotor carrier or the micromotor acidic environment. Overall, Fig. 2d verifies that the activity of the loaded drug was not compromised compared to free drug. Our in vitro results verified also that drug-loaded micromotors, made of Mg and other degradable materials, eventually destroy themselves and disappear in the acidic environment after releasing the CLR, with no apparent residues in the tissue. The findings validate the potential use of these drug-loaded micromotors for therapeutic applications.

### In vivo micromotor retention in mouse stomach

**Fig. 3:**
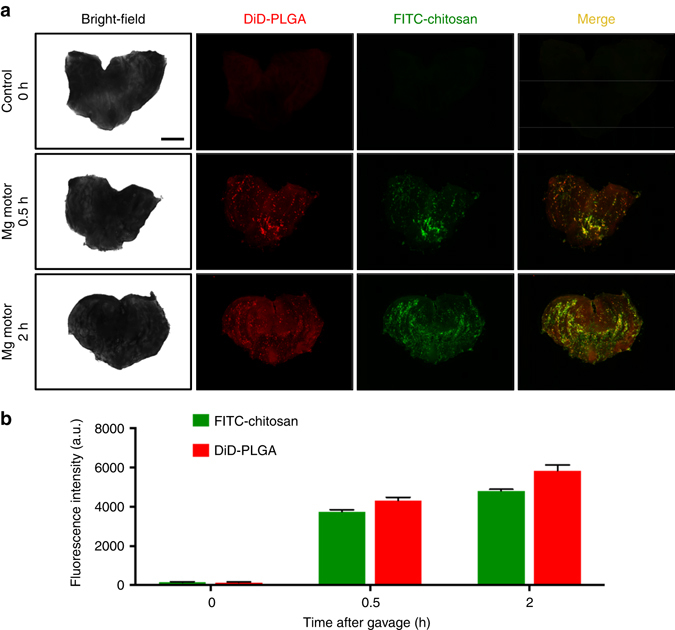
Retention of the Mg-based micromotors in mouse stomachs. **a** Bright-field and fluorescence images of the luminal lining of freshly excised mouse stomachs at 0 min after oral gavage of deionized (DI) water (control), and at 30 min and 2 h after oral gavage of the Mg-based micromotors. *Scale bar* 500 mm. **b** Corresponding fluorescence quantification of all the images shown in **a**. *Error bars* estimated as a triple of s.d. (*n* = 3)

After the optimization of drug-loading onto the Mg-based micromotors and the confirmation of effective in vitro bactericidal activity, the micromotors were further investigated under in vivo setting. First, the in vivo retention properties of the Mg-based micromotors on stomach tissue were examined at different post-administration times, and compared with control groups administered with DI water (Fig. 3). For this purpose, Mg-based micromotors prepared with DiD-labeled PLGA and FITC-labeled chitosan coatings were administered to a group of mice (*n* = 3), and following 30 min and 2 h of the samples administration, the mice were killed and the entire stomach was excised and opened. Subsequently, the luminal lining was rinsed with PBS and flattened for imaging. Accordingly, Fig. 3a shows bright-field and fluorescence images of the luminal lining of freshly excised mouse stomach at 0 min after oral gavage of DI water, and at 30 min and 2 h after oral gavage of Mg-based micromotors. As can be observed, the images corresponding to the dye-loaded Mg-based micromotors show an intense fluorescent signal in both *red* and *green* light channels, which indicates efficient distribution and retention of the micromotors in the mouse stomach. The continuous propulsion of the micromotors and the adhesive properties of the chitosan coating help to achieve a homogeneous distribution of the micromotors in the stomach. The corresponding fluorescence quantification of the dye-loaded micromotors retained in the mouse stomach after 30 min and 2 h oral gavage of the sample is displayed in Fig. 3b. The graphic represents the higher fluorescence signals obtained at 665 and 520 nm (corresponding to DiD and FITC dyes, respectively) for each sample. These results indicate that the micromotors can effectively propel in gastric fluid and are retained in the stomach wall, including the antrum, where the *H. pylori* bacteria reside. Such highly enhanced retention in the stomach, which is a major advantage of motor-enabled delivery, has been carefully examined in our early studies^22–24^. The powerful propulsion leads to tissue penetration and binding, so that the drug-loaded motor could reach the whole stomach wall for enhanced retention.

### In vivo anti-*H. pylori* therapeutic efficacy

**Fig. 4:**
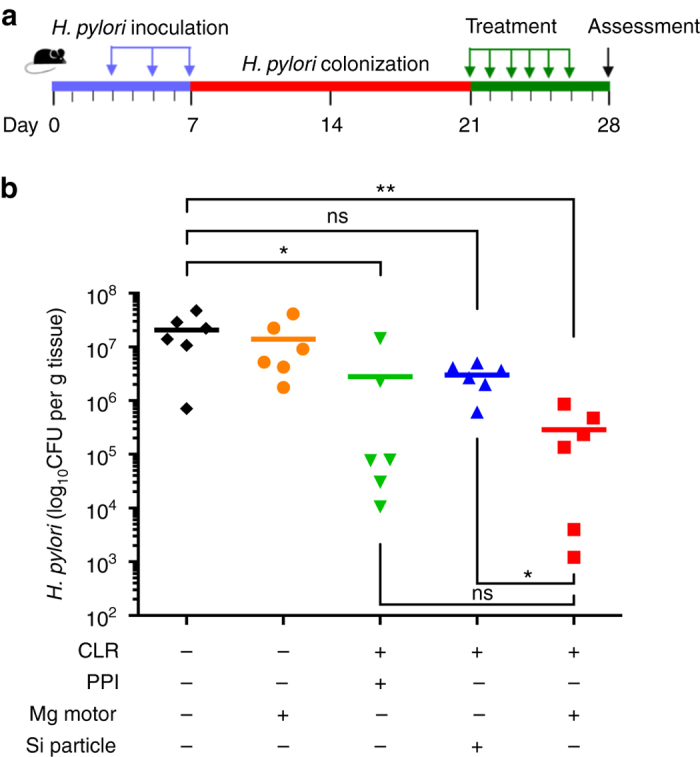
In vivo anti-*H. pylori* therapeutic efficacy. **a** The study protocol including *H. pylori* inoculation and infection development in C57BL/6 mice, followed by the treatments. **b** Quantification of bacterial burden in the stomach of *H. pylori*-infected mice treated with DI water (*black color*), bare Mg-based micromotors (*orange color*), free CLR+PPI (*green color*), CLR-loaded silica microparticles (*blue color*), and CLR-loaded Mg-based micromotors (*red color*), respectively (*n* = 6 per group). *Bars* represent median values. *P < 0.05, **P < 0.01, *ns* no statistical significance

We proceeded to test the in vivo therapeutic efficacy of the drug-loaded Mg-based micromotors against *H. pylori* infection. Prior to the therapeutic study, we developed *H. pylori* infection in a mouse model using C57BL/6 mice. Each mouse was inoculated with 3 × 10^8^ CFU *H. pylori* SS1 in brain–heart infusion (BHI) broth by oral gavage three times on day 3, 5, and 7 (Fig. 4a)^38, 39^. Two weeks after inoculation, the *H. pylori-*infected mice were divided into five groups (*n* = 6, for each group) and orally administered with DI water, blank Mg-based micromotors (without CLR drug), free CLR drug with PPI (CLR+PPI), CLR-loaded silica microparticles, or CLR-loaded Mg-based micromotors once a day for five consecutive days. On each day of treatment, mice in the free CLR+PPI group received 400 µmol kg^−1^ of omeprazole (as PPI treatment) 30 min before administrating CLR, to neutralize gastric acid and prevent potential degradation of CLR. Such PPI dosage has been reported to be effective both in reducing the gastric acidity in mouse models^40^, as well as in preserving the effectiveness of co-administered antibiotics^39, 41, 42^. After the treatment course, the bacterial burden was evaluated by enumerating and comparing *H. pylori* counts recovered from each mouse stomach. The mean bacterial burden from two negative control groups treated with DI water and blank Mg-based motors were 2.1 × 10^7^ and 1.4 × 10^7^ CFU g^−1^ of stomach tissue, respectively (Fig. 4b, *black* and *orange color*, respectively). Meanwhile, a bacterial burden of 3 × 10^6^ CFU g^−1^ was measured from the mice treated with CLR-loaded silica microparticles, which did not show statistical difference to the negative controls. In contrast, when the mice were treated with CLR-loaded Mg-based micromotors, the bacterial burden was quantified as 2.9 × 10^5^ CFU g^−1^, a significant reduction compared with the negative control and CLR-loaded silica microparticle groups. The substantial improvement in *H. pylori* reduction demonstrates the benefit of acid-powered Mg-based micromotors compared with static micron-sized carriers. A bacterial burden of 2.8 × 10^6^ CFU g^−1^ was obtained for the positive control mice with free CLR+PPI treatment. Although the difference between CLR-loaded Mg-based micromotors and the free CLR+PPI groups was not statistically significant, the CLR-loaded micromotors reduced the *H. pylori* burden in mice compared with in the negative controls by ~1.8 orders of magnitude, whereas the free CLR+PPI group reduced it only by ~0.8 orders of magnitude. These results might be derived from the benefit of the propulsion-enabled active drug delivery performed by the Mg-based micromotors in the stomach. These results demonstrate that the Mg-based micromotors can effectively propel and distribute throughout the stomach of living mice to significantly reduce *H. pylori* levels.

### In vivo toxicity evaluation of Mg-based micromotors

**Fig. 5:**
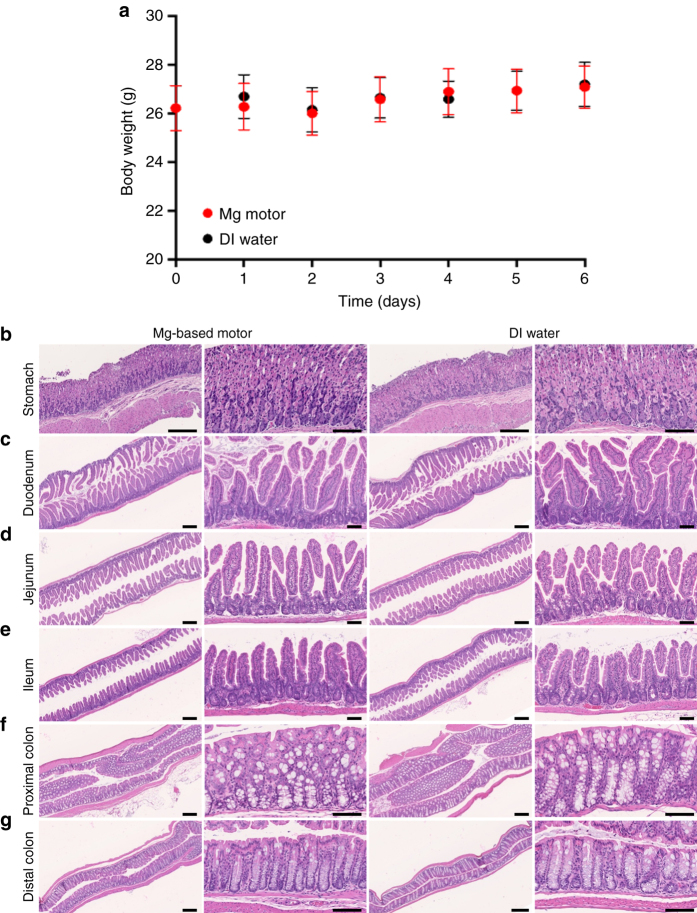
In vivo toxicity evaluation of the Mg-based micromotors. Uninfected mice were orally administered with the Mg-based micromotors or DI water once daily for five consecutive days. **a** Mouse body weight log from day 0 to day 6 of the toxicity study. *Error bars* represent the s.d. of the mean (*n* = 6). On day 6, mice were killed and sections of the mouse stomach **b**, small **c**–**e** and large **f**, **g** intestine tissues were processed for histological staining with hematoxylin and eosin (*H&E*). *Scale bars* Mg-motor, 250 and 100 μm (*left* and *right column*, respectively); DI water, 250 and 100 μm (*left* and *right column*, respectively)

Finally, the toxicity profile of the Mg-based micromotors in the stomach as well as in the lower GI tract was evaluated. Healthy mice were orally administered with Mg-based micromotors or DI water once daily for five consecutive days. Throughout the treatment, no signs of distress such as squinting of eyes, hunched posture, unkempt fur, or lethargy were observed in both groups. Initially, the toxicity profile of the Mg-micromotors in the mouse was evaluated through changes in body weight. During the experimental period, mice administered Mg-micromotors maintained a constant body weight compared with the mice administered DI water (Fig. 5a). On day 6, mice were killed and their stomachs and lower GI sections were processed for histological staining. Longitudinal sections of the glandular stomach (Fig. 5b), three major segments of small intestine (duodenum, jejunum, and ileum, Fig. 5c–e, respectively) and the two major segments of large intestine (proximal and distal colon, Fig. 5f–g, respectively) were stained with hematoxylin and eosin (H&E). The stomach and lower GI sections of the micromotor-treated group showed undamaged structure of columnar epithelial cells with no signs of superficial degeneration or erosion (Fig. 5b–g, *left*). There was no noticeable difference in the gastric and intestinal mucosal integrity, in terms of thickness as well as size and number of crypt and villus, between the motor-treated and DI water-treated groups (Fig. 5b–g, *left* vs. *right part*). No lymphocytic infiltration into the mucosa and submucosa was observed, indicating no sign of gastric inflammation. The in vivo toxicity studies of Mg-based micromotors showed no effect on the mouse body weight, apparent alteration of GI histopathology or observable inflammation, suggesting that the treatment of Mg-based micromotors is safe in the mouse model.

## Conclusions

In this work we conducted the first, to the best of our knowledge, study to evaluate the therapeutic efficacy of a drug-loaded Mg-based micromotor for in vivo treatment of *H. pylori* infection in a mouse model. Through these in vivo experiments, we demonstrated that acid-powered Mg-based micromotors could efficiently be loaded with clinical doses of drugs, retain in the mouse stomach wall, and perform an appreciable in vivo bactericidal activity. Our results showed that the active propulsion of drug-loaded Mg-based micromotors in the acidic media of the stomach and motor-tissue interaction lead to efficient drug delivery and hence to a significant reduction of bacteria burden compared to passive drug carriers. Furthermore, such drug-loaded micromotors function in gastric condition for the *H. pylori* infection treatment without the need of PPIs. We also demonstrated that there were no toxicological consequences of the micromotors in the mouse models. Overall, our results indicate that micromotors may be adapted to the development of new and safe therapeutic treatments against stomach diseases such as *H. pylori* infection. As our early studies have shown that the Mg-based micromotors can propel efficiently and position precisely in the GI tract^23, 24^, we believe the presented motor-enabled delivery approach is promising to treat diverse GI tract diseases. Extending the propulsion methods with new alternative biocompatible fuels^43, 44^ or fuel-free actuation^11–13^ might be able to expand the active-delivery concept to different parts of the body. We also envision that the micromotor approach will be useful for eliminating hard-to-treat bacterial biofilms,^45, 46^ with the efficient motor propulsion leading to biofilm penetration towards enhanced antibiotic delivery. Although the present results are promising, this work is still at its early stage. As a new active gastric delivery technology, future studies are required to further elucidate the micromotor’s in vivo delivery performance and functions, and to compare with other standard therapies against *H. pylori* infection or other gastric diseases. Nonetheless, this work opens the door to the use of synthetic motors as an active-delivery platform for in vivo treatment of diseases and will likely trigger intensive research interests in this area.

## Methods

### Synthesis of Mg-based micromotors

The Mg-based micromotors were prepared using magnesium (Mg) microparticles (catalog #FMW20, TangShan WeiHao Magnesium Powder Co.; average size, 20 ± 5 μm) as the core. The Mg microparticles were initially washed with acetone to eliminate the presence of impurities. After being dried under a N_2_ current, the Mg microparticles were dispersed onto glass slides (2 mg of Mg microparticles per glass slide), followed by ALD of TiO_2_ (at 100 °C for 120 cycles) using a Beneq TFS 200 system. As such an ALD process utilizes gas phase reactants, it leads to uniform coatings over the Mg microparticles, whereas still leaving a small opening at the contact point of the particle to the glass slide. After that, the Janus micromotors were coated with 120 µL of 1% (w/v) PLGA (Sigma-Aldrich, P2191) prepared in ethyl acetate (Sigma-Aldrich, 270989) and containing 40 mg mL^−1^ CLR (TCI CO., Ltd. C220). It should be noted that different CLR amounts (between 4 mg and 6 mg) were tested to optimize the drug-loading. The PLGA@CLR coating was dried fast to avoid crystallization of the drug. Finally, the Janus micromotors were coated with a thin layer of 0.05% (w/v) Chit (Sigma-Aldrich, C3646) prepared in water and containing 0.1% (w/v) sodium dodecyl sulfate (SDS) (Sigma-Aldrich, 62862) and 0.02% (v/v) acetic acid (Sigma-Aldrich, 695092), forming the outermost layer coated on the Mg microparticles. Finally, the Mg-based micromotors were collected by lightly scratching the microparticles off the glass slide.

### Synthesis of dye-loaded Mg-based micromotors

For performing the characterization of the Mg-based micromotors along with the in vivo retention studies, fluorescent Mg-based micromotors were prepared by combining both 1% PLGA and 0.05% Chit solutions with 5 µg mL^−1^ 1,1′-dioctadecyl-3,3,3′,3′-tetramethylindodicarbocyanine, 4-chlorobenzenesulfonate salt (DiD, *λ*_ex_ = 644 nm/*λ*_em_ = 665 nm, Life Technologies, D7757) and 1 µg mL^−1^ fluorescein isothiocyanate-dextran (FITC, *λ*_ex_ = 492 nm/*λ*_em_ = 520 nm, Sigma-Aldrich, 46945) dyes, respectively. To compare with the Mg-based micromotors, inert silica (Si) microparticles (Nanocs, Inc., Cat. No. Si01-20u-1; 20 µm size) were used as core particles, following the same protocol described above.

### Micromotor characterization

Bright-field and fluorescent images of the Mg-based micromotors and inert silica microparticles (Supplementary Fig. 2) were captured using a EVOS FL microscope coupled with a ×20 and ×40 microscope objectives and fluorescence filters for *red* and *green* light excitation.

Scanning electron microscopy (SEM) images of the Mg-based micromotors were obtained with a Phillips XL30 ESEM instrument, using an acceleration voltage of 10 kV. EDX mapping analysis was performed using an Oxford EDX detector attached to SEM instrument and operated by INCA software.

### Micromotor propulsion studies

Autonomous Mg-based micromotors propulsion in simulated gastric fluid (Sigma-Aldrich, 01651) was obtained by diluting 25 times the simulated gastric fluid according to the commercial specifications (final pH ~1.3), and adding 1% Triton X-100 (Fisher Scientific, FairLawn, NJ, USA) as surfactant. An inverted optical microscope (Nikon Eclipse 80i upright microscope) coupled with different microscope objectives (×10, ×20, and ×40) and a QuantEM:512SC camera were used for recording the autonomous micromotor propulsion in the gastric fluid simulant. The speed of the Mg-based micromotors was characterized using the MetaMorph 7.1 software (Molecular Devices, Sunnyvale, CA, USA).

### In vitro anti-*H. pylori* activity

*H. pylori* Sydney strain 1 (HPSS1) was cultured from frozen stock and routinely maintained on Columbia agar supplemented with 5% (vol/vol) laked horse blood at 37 °C under microaerobic conditions (10% CO_2_, 85% N_2_, and 5% O_2_). For experiments, broth cultures of *H. pylori* were prepared by subculturing fresh colonies from agar plates into brain–heart infusion (BHI) supplemented with 5% fetal bovine serum (FBS) and incubated overnight at 37 °C under microaerobic conditions with moderate reciprocal shaking. An overnight broth culture of *H. pylori* was centrifuged at 5000×*g* for 10 min to obtain a bacterial pellet. After removal of culture medium by centrifugation, the obtained bacteria pellet was then suspended in an appropriate amount of fresh BHI with 5% FBS for future use.

The bactericidal activity against *H. pylori* of free CLR and CLR-loaded Mg-based micromotors (PLGA@CLR-TiO_2_-Mg) were tested in vitro. All samples were treated in 0.1 N HCl for 1 h and serially diluted to desired concentrations with PBS (pH 7). Bare Mg-based micromotors (PLGA-TiO_2_-Mg) with corresponding amount of micromotors were used as negative control.

The samples were added with 1 × 10^6^ CFU mL^−1^ *H. pylori* in BHI with 5% FBS to make final concentrations of 0–16 μg mL^−1^ CLR, followed by incubation at 37 °C under microaerobic conditions with moderate reciprocal shaking for 24 h. Then, a series of 10-fold dilutions of the bacterial suspension was prepared, and inoculated onto a Columbia agar plates supplemented with 5% laked horse blood. The plates were cultured for 4 days before the colony-forming unit (CFU) of *H. pylori* was quantified. All measurements were made in triplicate.

### In vivo micromotor retention

Prior to the experiment, C57BL/6 mice (*n* = 3) were fed with alfalfa-free food from LabDiet (St Louis, MO, USA) for 2 weeks. The in vivo retention study was performed by using dye-loaded Mg-based micromotors prepared by the protocol described above. A 0.3 mL suspension of Mg-based micromotors with DiD-labeled PLGA and FITC-labeled chitosan coatings were intragastrically administered. A group of mice was administered with DI water as a negative control. Following 30 min and 2 h of oral administrations, the mice were killed and their entire stomachs were excised and cut opened along the greater curvature. Then, the tissues were rinsed with PBS, flattened, and visualized using a Keyence BZ-X700 fluorescence microscope. The bright-field and corresponding fluorescence images were obtained at 665 and 520 nm (DiD and FITC, respectively) for each sample. Subsequently, the tissues were transferred to 1 mL PBS and homogenized. Analysis of the amount of micromotors retained in the stomachs was carried out by measuring the fluorescence intensity of their embedded DiD-labeled PLGA and FITC-labeled chitosan using Synergy Mx fluorescent spectrophotometer (Biotek, Winooski, VT, USA). All animal experiments were in compliance with the University of California San Diego Institutional Animal Care and Use Committee (IACUC) regulations.

### In vivo therapeutic efficacy against *H. pylori* infection

Six-week-old C57BL/6 male mice were purchased from the Jackson Laboratory (Bar Harbor, ME, USA). Each C57BL/6 mouse received 0.3 mL of 1 × 10^9^ CFU mL^−1^ *H. pylori* in BHI broth administered intragastrically through oral gavage every 48 h, repeated three times (on day 3, 5, and 7, respectively), and the infection was allowed to develop for 2 weeks^39^. For the in vivo anti-*H. pylori* therapeutic study, mice were randomly divided in five treatment groups (*n* = 6) to be orally administered once daily for five consecutive days with CLR-loaded Mg-based micromotors, CLR-loaded inert silica microparticles, free CLR+PPI, blank Mg-based micromotors or DI water. For free CLR+PPI group, each day of treatment mice were first administered with omeprazole (a PPI) through oral gavage at a dose of 400 μmol kg^−1^ ^39, 40–42^, followed by a lag time of 30 min before administration of CLR. CLR-loaded Mg-based micromotors, CLR-loaded inert silica microparticles and free CLR (with 30 mg kg^−1^ CLR dosage) were also administered through oral gavage once daily for five consecutive days^39^. Blank Mg-based micromotors and DI water served as movement control and negative control, respectively. Forty-eight hours after last administration^39, 47–49^, mice were killed and stomachs were excised from the abdominal cavity. The stomachs were cut along the greater curvature, and the gastric content were removed and rinsed with PBS. For *H. pylori* recovery, each gastric tissue was weighed before suspended in 200 μL PBS and homogenized. The homogenate was serially diluted and spotted onto Columbia agar plate with 5% laked horse blood and Skirrow’s supplement (10 μg mL^−1^ vancomycin, 5 μg mL^−1^ trimethoprim lactate, and 2500 IU/L polymyxin B; Oxiod). The plates were then incubated at 37 °C under microaerobic conditions for 5 days, and bacterial colonies were enumerated. Statistical analysis was performed using one-way ANOVA. No statistical methods were used to predetermine sample size. Studies were done in a non-blinded fashion. Replicates represent different mice subjected to the same treatment (*n* = 6). All animal experiments were in compliance with the University of California San Diego Institutional Animal Care and Use Committee (IACUC) regulations.

### Toxicity evaluation of Mg-based micromotors

To evaluate the acute toxicity of the Mg-based micromotors in vivo, uninfected C57BL/6 male mice (*n* = 6) weighing 25–30 g were orally administered with CLR-loaded micromotors once daily for five consecutive days. Mice administered with DI water were tested in parallel as a negative control. During the experimental period, the mouse body weight was monitored by weighing the mice daily. On day 6, mice were killed and sections of the mouse stomach, small and large intestine tissues were processed for histological examination. The stomach was cut open along the greater curvature, and the gastric content was removed. The small and large intestines were cut to small sections as duodenum, jejunum, ileum, proximal, and distal colon and rinsed inside with PBS to remove internal residues. The longitudinal tissue sections were fixed in neutral-buffered 10% (vol/vol) formalin for 15 h, transferred into 70% ethanol, and embedded in paraffin. The tissue sections were cut with 5 μm thickness and stained with H&E assay. The stained sections were visualized by Hamamatsu NanoZoomer 2.0HT and the images processed using NDP viewing software. All animal experiments were in compliance with the University of California San Diego Institutional Animal Care and Use Committee (IACUC) regulations.

### Data availability

The authors declare that all relevant data supporting the findings of this study are available within the article and its supplementary information files as well from the authors upon reasonable request.

## Electronic supplementary material

Supplementary Information

Supplementary Movie 1

Supplementary Movie 2

Supplementary Movie 3

Supplementary Movie 4
