# Author correction: Micromotor-enabled active drug delivery for in vivo treatment of stomach infection

**DOI:** 10.1038/s41467-017-01616-y

**Published:** 2017-10-31

**Authors:** Berta Esteban-Fernández de Ávila, Pavimol Angsantikul, Jinxing Li, Miguel Angel Lopez-Ramirez, Doris E. Ramírez-Herrera, Soracha Thamphiwatana, Chuanrui Chen, Jorge Delezuk, Richard Samakapiruk, Valentin Ramez, Marygorret Obonyo, Liangfang Zhang, Joseph Wang

**Affiliations:** 1 Department of NanoEngineering, University of California San Diego, La Jolla, CA 92093 USA; 2 Department of Medicine, University of California San Diego, La Jolla, CA 92093 USA

*Nature Communications* **8**:272 doi:10.1038/s41467-017-00309-w; Article published online 16 August 2017

Marygorret Obonyo, who provided the *H. pylori* SS1 strain for this work and participated in the design of *H. pylori* infection studies, was inadvertently omitted from the author list. This has now been corrected in both the PDF and HTML versions of the Article.

