# Supplementary material for: Micromotor-enabled active drug delivery for in vivo treatment of stomach infection
Source: Nat Commun. 2017 Aug 16;8:272. doi: 10.1038/s41467-017-00309-w (PMC5559609; doi:10.1038/s41467-017-00309-w)
Supplement: Supplementary file 1 — Supplementary Information [file 41467_2017_309_MOESM1_ESM.pdf]

File Name: Supplementary Information
Description: Supplementary Figures

File Name: Supplementary Movie 1
Description: Preparation protocol of the drug-loaded Mg-based micromotors.

File Name: Supplementary Movie 2
Description: In vivo self-propulsion of drug-loaded Mg-based micromotors in the gastric fluid of the stomach.

File Name: Supplementary Movie 3
Description: Autonomous movement of drug-loaded Mg-based micromotors in simulated gastric fluid.

File Name: Supplementary Movie 4
Description: Movement of drug-loaded Mg-based micromotors in simulated gastric fluid adjusted to different pHs.

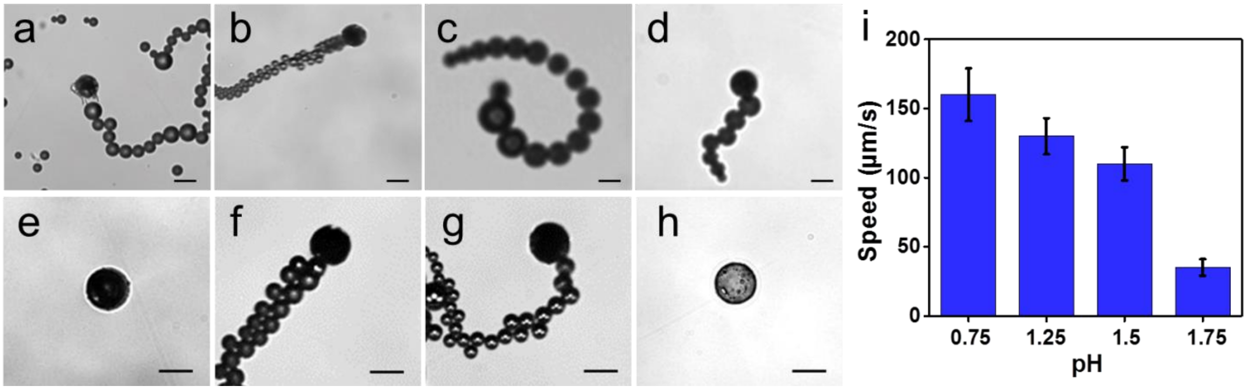

Supplementary Figure 1. Propulsion characterization of the drug-loaded Mg-based micromotors. Time-lapse images (corresponding to Supplementary Movie 4) showing the motion of the drug-loaded Mg-based micromotors in (**a**) pH 0.75, (**b**) pH 1.25, (**c**) pH 1.5, and (**d**) pH 1.75. Time-lapse images showing the lifetime of the drug-loaded Mg-based micromotor in simulated gastric fluid (pH ~1.3); from **e** to **h**: 0, 1, 5 and 8 min after gastric fluid simulant addition, respectively. (**i**) Dependence of the micromotor speed upon the gastric fluid pH. Scale bar, 20 μm. Surfactant level, 1% Triton X-100. Error bars estimated as a triple of s.d. (n = 6).

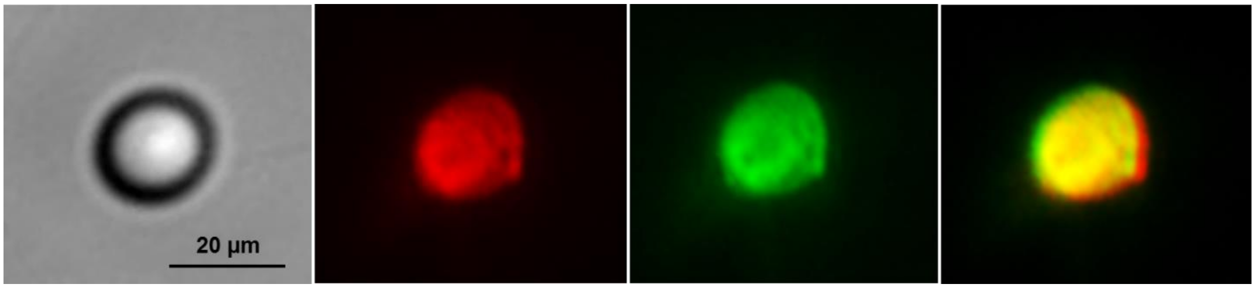

Supplementary Figure 2. Characterization of dye-loaded chitosan-PLGA-silica Janus microparticles. Microscopy images of dye-loaded chitosan-PLGA-Si Janus microparticles: optical image, and fluorescence images showing the dye-loaded Si-based Janus microparticles in DiD channel, FITC-D channel, and overlay of the two channels.
